# Supplementary material for: Pooled incidence and case-fatality of acute stroke in Mainland China, Hong Kong, and Macao: A systematic review and meta-analysis
Source: PLoS One. 2022 Jun 27;17(6):e0270554. doi: 10.1371/journal.pone.0270554 (PMC9236238; doi:10.1371/journal.pone.0270554)
Supplement: S1 Table — (DOCX) [file pone.0270554.s002.docx]

**S1 Table(a). Search strategy (PubMed)**

| # | Searches |
| --- | --- |
| 1 | "China"[Mesh] OR China OR Anhui OR Fujian OR Gansu OR Guangdong OR Guizhou OR Hainan OR Hebei OR Heilongjiang OR Henan OR Hubei OR Hunan OR Jiangsu OR Jiangxi OR Jilin OR Liaoning OR Qinghai OR Shaanxi OR Shandong OR Shanxi OR Sichuan OR Yunnan OR Zhejiang OR Guangxi OR Inner Mongolia OR Nei Menggu OR Neimenggu OR Ningxia OR Xinjiang OR Tibet OR Xizang OR Beijing OR Chongqing OR Shanghai OR Tianjin OR Hong Kong OR Xianggang OR Macau OR Macao OR Aomen OR People's Republic of China OR PRC |
| 2 | "Stroke"[Mesh] OR "Stroke, Lacunar"[Mesh] OR "Stroke Rehabilitation"[Mesh] OR "Brain Stem Infarctions"[Mesh] OR "Brain Ischemia"[Mesh] OR "Hypoxia-Ischemia, Brain"[Mesh] OR "Brain Injuries"[Mesh] OR "Cerebral Infarction"[Mesh] OR "Infarction, Anterior Cerebral Artery"[Mesh] OR "Infarction, Middle Cerebral Artery"[Mesh] OR "Infarction, Posterior Cerebral Artery"[Mesh] OR "Intracranial Embolism and Thrombosis"[Mesh] OR "Intracranial Hemorrhages"[Mesh] OR "Intracranial Hemorrhage, Hypertensive"[Mesh] OR "Vertebro-Basilar Ischemia"[Mesh] OR "Ischemia, Vertebro-Basilar"[Mesh] OR "Ischemias, Vertebro-Basilar"[Mesh] OR "Vertebro Basilar Ischemia"[Mesh] OR "Vertebro-Basilar Ischemias"[Mesh] OR "Vertebrobasilar Ischemia"[Mesh] OR "Ischemia, Vertebrobasilar"[Mesh] OR "Ischemias, Vertebrobasilar"[Mesh] OR "Vertebrobasilar Ischemias"[Mesh] OR "Artery Ischemia, Vertebral"[Mesh] OR "Artery Ischemias, Vertebral"[Mesh] OR "Ischemia, Vertebral Artery"[Mesh] OR "Ischemias, Vertebral Artery"[Mesh] OR "Vertebral Artery Ischemias"[Mesh] OR "Basilar Artery Ischemia"[Mesh] OR "Artery Ischemia, Basilar"[Mesh] OR "Artery Ischemias, Basilar"[Mesh] OR "Basilar Artery Ischemias"[Mesh] OR "Ischemia, Basilar Artery"[Mesh] OR "Ischemias, Basilar Artery"[Mesh] OR "Artery Dissection, Vertebral"[Mesh] OR "Artery Dissections, Vertebral"[Mesh] OR "Dissection, Vertebral Artery"[Mesh] OR "Dissections, Vertebral Artery"[Mesh] OR "Vertebral Artery Dissections"[Mesh] OR "Dissecting Vertebral Artery Aneurysm"[Mesh] OR "Spontaneous Vertebral Artery Dissection"[Mesh] OR "Vertebral Artery Dissection, Spontaneous"[Mesh] OR "Dissection, Internal Carotid Artery"[Mesh] OR "Internal Carotid Artery Dissection"[Mesh] OR "Carotid Artery Dissection, Internal"[Mesh] OR "Thrombosis, Carotid"[Mesh] OR "Thrombosis, Carotid Artery"[Mesh] OR "Carotid Artery Thromboses"[Mesh] OR "Carotid Thrombosis"[Mesh] OR "External Carotid Artery Thrombosis"[Mesh] OR "Thrombosis, External Carotid Artery"[Mesh] OR "Internal Carotid Artery Thrombosis"[Mesh] OR "Thrombosis, Internal Carotid Artery"[Mesh] OR "Common Carotid Artery Thrombosis"[Mesh] OR "Thrombosis, Common Carotid Artery"[Mesh] OR "Cerebral Hemorrhage"[Mesh] OR "Subarachnoid Hemorrhage"[Mesh] OR "Basal Ganglia Hemorrhage"[Mesh] OR "Cerebral Intraventricular Hemorrhage"[Mesh] OR "Putaminal Hemorrhage"[Mesh] OR "Hematoma, Subdural"[Mesh] |
|  |  |
| 3 | stroke*[Title/Abstract] OR apoplexia[Title/Abstract] OR apoplexy[Title/Abstract] OR brain attack*[Title/Abstract] OR brain ischemia*[Title/Abstract] OR brain ischaemia*[Title/Abstract] OR ischemic attack*[Title/Abstract] OR ischaemic attack*[Title/Abstract] OR ischemic event*[Title/Abstract] OR ischaemic event*[Title/Abstract] OR brain infarct*[Title/Abstract] OR cerebral infarct*[Title/Abstract] OR cerebral ischemia*[Title/Abstract] OR cerebral ischaemia*[Title/Abstract] OR cerebell* infarct*[Title/Abstract] OR cerebell* ischemia*[Title/Abstract] OR cerebell* ischaemia*[Title/Abstract] OR cerebral hemorrhag*[Title/Abstract] OR cerebral haemorrhag*[Title/Abstract] OR intracerebral hemorrhag*[Title/Abstract] OR intracerebral haemorrhag*[Title/Abstract] OR intracranial hemorrhage[Title/Abstract] OR intracranial haemorrhag*[Title/Abstract] OR subdural hemorrhag*[Title/Abstract] OR subdural haemorrhag*[Title/Abstract] OR subarachnoid hemorrhag*[Title/Abstract] OR subarachnoid haemorrhag*[Title/Abstract] OR cerebell* hemorrhag*[Title/Abstract] OR cerebell* haemorrhag*[Title/Abstract] OR cerebell* haematoma*[Title/Abstract] OR cerebell* hematoma*[Title/Abstract] OR cerebral haematoma*[Title/Abstract] OR cerebral hematoma*[Title/Abstract] OR brain haematoma*[Title/Abstract] OR brain hematoma*[Title/Abstract] OR cerebell* bleed*[Title/Abstract] OR cerebral bleed*[Title/Abstract] OR brain* bleed*[Title/Abstract] OR ruptured brain aneurysm[Title/Abstract] OR ruptured cerebral aneurysm[Title/Abstract] OR ruptured brain aneurism[Title/Abstract]  OR ruptured cerebral aneurism[Title/Abstract] OR cerebrovascular[Title/Abstract] OR cerebral vascular[Title/Abstract]  OR cva[Title/Abstract] OR cerebrovascular accident*[Title/Abstract] OR brain injur*[Title/Abstract] OR poststroke[Title/Abstract] OR post stroke[Title/Abstract] OR hemiplegia[Title/Abstract] OR paresis[Title/Abstract] OR dystonia[Title/Abstract] |
| 4 | #2 or #3 |
| 5 | "Incidence"[Mesh] OR "Epidemiology"[Mesh] OR "Morbidity"[Mesh] OR "Public Health Surveillance"[Mesh] OR "Sentinel Surveillance"[Mesh] OR "Population Surveillance"[Mesh] OR "Epidemiological Monitoring"[Mesh] OR "Epidemiologic Studies"[Mesh] OR "Epidemiologic Research Design"[Mesh] OR "Epidemiologic Methods"[Mesh] |
| 6 | incidence OR incident OR frequency OR occurrence OR morbidit* OR surveillance OR epidemiolog* OR attack rate* |
| 7 | #5 or #6 |
| 8 | #1 and #4 and #7 |
| 9 | #1 and #4 and #7 Filters: Humans |
| 10 | #1 and #4 and #7 Filters: Humans; English |
| 11 | #1 and #4 and #7 Filters: Publication date from 1990/01/01; Humans; English |
| 12 | "Mortality"[Mesh] OR "Mortality, Premature"[Mesh] OR "Hospital Mortality"[Mesh] OR "Death"[Mesh] OR "Cause of Death"[Mesh] OR "Death, Sudden"[Mesh] OR "Brain Death"[Mesh] OR "Death Certificates"[Mesh] OR "Karoshi Death"[Mesh] |
| 13 | mortal*[Title/Abstract] OR death*[Title/Abstract] OR dead[Title/Abstract] OR died[Title/Abstract] OR fatality[Title/Abstract] OR case fatality[Title/Abstract] |
| 14 | #12 or #13 |
| 15 | #8 and #14 |
| 16 | #8 and #14 Filters: Humans |
| 17 | #8 and #14 Filters: Humans; English |
| 18 | #8 and #14 Filters: Publication date from 1990/01/01; Humans; English |

**S1 Table(b). Search strategy (SinoMed)**

| # | Searches |
| --- | --- |
| 1 | "中国"[不加权:扩展] OR "安徽"[不加权:扩展] OR "福建"[不加权:扩展] OR "甘肃"[不加权:扩展] OR "广东"[不加权:扩展] OR "贵州"[不加权:扩展] OR "海南"[不加权:扩展] OR "河北"[不加权:扩展] OR "黑龙江"[不加权:扩展] OR "河南"[不加权:扩展] OR "湖北"[不加权:扩展] OR "湖南"[不加权:扩展] OR "江苏"[不加权:扩展] OR "江西"[不加权:扩展] OR "吉林"[不加权:扩展] OR "辽宁"[不加权:扩展] OR "青海"[不加权:扩展] OR "陕西"[不加权:扩展] OR "山东"[不加权:扩展] OR "山西"[不加权:扩展] OR "四川"[不加权:扩展] OR "云南"[不加权:扩展] OR "浙江"[不加权:扩展] OR "广西[壮族自治区]"[不加权:扩展] OR "内蒙古[自治区]"[不加权:扩展] OR "宁夏[回族自治区]"[不加权:扩展] OR "新疆[维吾尔自治区]"[不加权:扩展] OR "西藏[自治区]"[不加权:扩展] OR "北京"[不加权:扩展] OR "重庆"[不加权:扩展] OR "上海"[不加权:扩展] OR "天津"[不加权:扩展] OR "香港"[不加权:扩展] OR "澳门"[不加权:扩展] |
| 2 | "中国"[全部字段:智能] OR "安徽"[全部字段:智能] OR "福建"[全部字段:智能] OR "甘肃"[全部字段:智能] OR "广东"[全部字段:智能] OR "贵州"[全部字段:智能] OR "海南"[全部字段:智能] OR "河北"[全部字段:智能] OR "黑龙江"[全部字段:智能] OR "河南"[全部字段:智能] OR "湖北"[全部字段:智能] OR "湖南"[全部字段:智能] OR "江苏"[全部字段:智能] OR "江西"[全部字段:智能] OR "吉林"[全部字段:智能] OR "辽宁"[全部字段:智能] OR "青海"[全部字段:智能] OR "陕西"[全部字段:智能] OR "山东"[全部字段:智能] OR "山西"[全部字段:智能] OR "四川"[全部字段:智能] OR "云南"[全部字段:智能] OR "浙江"[全部字段:智能] OR "广西"[全部字段:智能] OR "内蒙古"[全部字段:智能] OR "宁夏"[全部字段:智能] OR "新疆"[全部字段:智能] OR "西藏"[全部字段:智能] OR "北京"[全部字段:智能] OR "重庆"[全部字段:智能] OR "上海"[全部字段:智能] OR "天津"[全部字段:智能] OR "香港"[全部字段:智能] OR "澳门"[全部字段:智能] OR "中华人民共和国"[全部字段:智能] |
|  |  |
| 3 | (#2) OR (#1) |
| 4 | "卒中"[不加权:扩展] OR "中风, 腔隙性"[不加权:扩展] OR "中风康复"[不加权:扩展] OR "脑干梗死"[不加权:扩展] OR "脑缺血"[不加权:扩展] OR "缺氧缺血, 脑"[不加权:扩展] OR "脑损伤"[不加权:扩展] OR "大脑梗死"[不加权:扩展] OR "梗死, 大脑前动脉"[不加权:扩展] OR "梗死, 大脑中动脉"[不加权:扩展] OR "梗死, 大脑后动脉"[不加权:扩展] OR "颅内栓塞和血栓形成"[不加权:扩展] OR "颅内出血"[不加权:扩展] OR "颅内出血, 高血压性"[不加权:扩展] OR "椎底动脉供血不足"[不加权:扩展] OR "椎动脉破裂"[不加权:扩展] OR "颈内动脉夹层"[不加权:扩展] OR "颈动脉血栓形成"[不加权:扩展] OR "脑出血"[不加权:扩展] OR "蛛网膜下腔出血"[不加权:扩展] OR "脑血管基底神经节出血"[不加权:扩展] OR "壳核出血"[不加权:扩展] OR "血肿, 硬膜下"[不加权:扩展] |
| 5 | "卒中"[中文标题:智能] OR "卒中"[摘要:智能] OR "脑卒中"[中文标题:智能] OR "脑卒中"[摘要:智能] OR "中风"[中文标题:智能] OR "中风"[摘要:智能] OR "脑中风"[中文标题:智能] OR "脑中风"[摘要:智能] OR "脑血管中风"[中文标题:智能] OR "脑血管中风"[摘要:智能] OR "脑缺血"[中文标题:智能] OR "脑缺血"[摘要:智能] OR "脑缺血发作"[中文标题:智能] OR "脑缺血发作"[摘要:智能] OR "脑缺血事件"[中文标题:智能] OR "脑缺血事件"[摘要:智能] OR "脑梗塞"[中文标题:智能] OR "脑梗塞"[摘要:智能] OR "脑梗"[中文标题:智能] OR "脑梗"[摘要:智能] OR "脑梗死"[中文标题:智能] OR "脑梗死"[摘要:智能] OR "脑出血"[中文标题:智能] OR "脑出血"[摘要:智能] OR "大脑出血"[中文标题:智能] OR "大脑出血"[摘要:智能] OR "脑溢血"[中文标题:智能] OR "脑溢血"[摘要:智能] OR "大脑溢血"[中文标题:智能] OR "大脑溢血"[摘要:智能] OR "脑内出血"[中文标题:智能] OR "脑内出血"[摘要:智能] OR "颅内出血"[中文标题:智能] OR "颅内出血"[摘要:智能] OR "硬脑膜下出血"[中文标题:智能] OR "硬脑膜下出血"[摘要:智能] OR "硬膜下出血"[中文标题:智能] OR "硬膜下出血"[摘要:智能] OR "蛛网膜下腔出血"[中文标题:智能] OR "蛛网膜下腔出血"[摘要:智能] OR "蛛网膜下出血"[中文标题:智能] OR "蛛网膜下出血"[摘要:智能] OR "小脑出血"[中文标题:智能] OR "小脑出血"[摘要:智能] OR "小脑溢血"[中文标题:智能] OR "小脑溢血"[摘要:智能] OR "脑血肿"[中文标题:智能] OR "脑血肿"[摘要:智能] OR "脑动脉瘤破裂"[中文标题:智能] OR "脑动脉瘤破裂"[摘要:智能] OR "脑血管"[中文标题:智能] OR "脑血管"[摘要:智能] OR "脑血管意外"[中文标题:智能] OR "脑血管意外"[摘要:智能] OR "脑损伤"[中文标题:智能] OR "脑损伤"[摘要:智能] OR "卒中后"[中文标题:智能] OR "卒中后"[摘要:智能] OR "脑卒中后"[中文标题:智能] OR "脑卒中后"[摘要:智能] OR "脑中风后"[中文标题:智能] OR "脑中风后"[摘要:智能] OR "偏瘫"[中文标题:智能] OR "偏瘫"[摘要:智能] OR "半身麻痹"[中文标题:智能] OR "半身麻痹"[摘要:智能] OR "半身不遂"[中文标题:智能] OR "半身不遂"[摘要:智能] OR "轻度瘫痪"[中文标题:智能] OR "轻度瘫痪"[摘要:智能] OR "轻瘫"[中文标题:智能] OR "轻瘫"[摘要:智能] OR "不全麻痹"[中文标题:智能] OR "不全麻痹"[摘要:智能] OR "不全性麻痹"[中文标题:智能] OR "不全性麻痹"[摘要:智能] OR "局部麻痹"[中文标题:智能] OR "局部麻痹"[摘要:智能] OR "肌张力障碍"[中文标题:智能] OR "肌张力障碍"[摘要:智能] OR "肌张力失常"[中文标题:智能] OR "肌张力失常"[摘要:智能] |
| 6 | (#5) OR (#4) |
| 7 | "发病率"[不加权:扩展] OR "流行病学"[不加权:扩展] OR "年患病率"[不加权:扩展] OR "公共卫生监测"[不加权:扩展] OR "哨点监测"[不加权:扩展] OR "人群监测"[不加权:扩展] OR "流行病学监测"[不加权:扩展] OR "流行病学研究"[不加权:扩展] OR "流行病学研究设计"[不加权:扩展] OR "流行病学方法"[不加权:扩展] |
| 8 | "发病率"[全部字段:智能] OR "发生率"[全部字段:智能] OR "监测"[全部字段:智能] OR "流行病"[全部字段:智能] OR "罹患率"[全部字段:智能] |
| 9 | (#8) OR (#7) |
| 10 | (#9) AND (#6) AND (#3) |
| 11 | ((#9) AND (#6) AND (#3)) AND ( 人类[特征词]) AND 1990-2021[日期] |
| 12 | (((#9) AND (#6) AND (#3)) AND ( 人类[特征词]) AND 1990-2021[日期]) AND ("1"[期刊类型]) |
| 13 | "死亡率"[不加权:扩展] OR "死亡率, 过早"[不加权:扩展] OR "医院死亡率"[不加权:扩展] OR "死亡"[不加权:扩展] OR "死亡原因"[不加权:扩展] OR "猝死"[不加权:扩展] OR "脑死亡"[不加权:扩展] OR "死亡证"[不加权:扩展] OR "过劳死"[不加权:扩展] |
| 14 | "死亡率"[中文标题:智能] OR "死亡率"[摘要:智能] OR "病死率"[中文标题:智能] OR "病死率"[摘要:智能] OR "死亡"[中文标题:智能] OR "死亡"[摘要:智能] OR "逝世"[中文标题:智能] OR "逝世"[摘要:智能] OR "去世"[中文标题:智能] OR "去世"[摘要:智能] |
| 15 | (#14) OR (#13) |
| 16 | (#15) AND (#10) |
| 17 | ((#15) AND (#10)) AND ( 人类[特征词]) AND 1990-2021[日期] |
| 18 | (((#15) AND (#10)) AND ( 人类[特征词]) AND 1990-2021[日期]) AND ("1"[期刊类型]) |
