# Supplementary material for: Pooled incidence and case-fatality of acute stroke in Mainland China, Hong Kong, and Macao: A systematic review and meta-analysis
Source: PLoS One. 2022 Jun 27;17(6):e0270554. doi: 10.1371/journal.pone.0270554 (PMC9236238; doi:10.1371/journal.pone.0270554)
Supplement: S2 Table — (DOCX) [file pone.0270554.s003.docx]

**S2 Table(a). Risk of bias assessments (incidence studies)**

| Study | Selection | Comparability | Outcome | AHRQ standards |
| --- | --- | --- | --- | --- |
| Du et al, 1997 | ★★ | ★ | ★★★ | fair |
| Duan et al, 2019 | ★★ | ★ | ★★ | fair |
| Gu et al, 2020 | ★ | ★ | ★ | poor |
| Guo et al, 2013 | ★★★ | ★ | ★★ | good |
| Jia et al, 2011 | ★★ | ★ | ★ | poor |
| Li et al, 2015 | ★★ | ★ | ★ | poor |
| Liu et al, 2014 | ★★ | ★ | ★★ | fair |
| Wang et al, 2016 | ★★★ | ★ | ★★★ | good |
| Wang et al, 2018 | ★★★ | ★ | ★★ | fair |
| Wang et al, 2019 | ★★ | ★ | ★★★ | fair |
| Yu et al, 2016 | ★★ | ★★ | ★★★ | fair |
| Zhang et al, 2019 | ★★ | ★ | ★★ | fair |
| Ji et al, 2020 | ★★ | ★★ | ★ | Poor |
| Wang et al, 2020 | ★★ | ★★ | ★ | Poor |
| Han et al, 2017 | ★★ | ★ | ★★ | fair |
| Zhou et al, 2020 | ★★ | ★★ | ★★ | fair |
| Li et al, 2018 | ★★ | ★ | ★ | poor |
| Sun et al, 2013 | ★★ | ★ | ★★★ | fair |
| Xie et al, 2019 | ★★ | ★ | ★★★ | fair |
| Yu et al, 2020 | ★★ | ★★ | ★★ | fair |
| Guo et al, 2016 | ★★ | ★ | ★ | poor |
| Olofindayo et al, 2015 | ★★ | ★ | ★★★ | fair |
| Ren et al, 2019 | ★★ | ★ | ★ | poor |
| Zhang et al, 2009 | ★★ | ★ | ★★ | fair |
| Zhou et al, 2016 | ★★ |  | ★ | poor |
| Huang et al, 2003 | ★★★ |  | ★★★ | poor |
| Huang et al, 2019 | ★★★★ | ★ | ★★★★ | good |
| Kelly et al, 2008 | ★★★★ | ★★ | ★★★ | good |
| Liu et al, 2008 | ★ | ★ | ★ | poor |
| Wang et al, 2013 | ★★ | ★★ | ★★ | fair |
| Zhang et al, 2007 | ★ | ★ | ★★ | poor |
| Zhou et al, 2003 | ★★ | ★ | ★★★ | fair |
| Chen et al, 2020 | ★★ | ★★ | ★★★★ | fair |
| Swaminathan et al, 2020 | ★★ | ★★ | ★★ | fair |

**S2 Table(b). Risk of bias assessments (case fatality studies)**

| Study | Selection | Comparability | Outcome | AHRQ standards |
| --- | --- | --- | --- | --- |
| Liu et al, 2018 | ★★ |  | ★★ | poor |
| Shi at al, 2018 | ★★★ | ★★ | ★★ | good |
| Geng et al, 2016 | ★★ | ★ | ★★ | fair |
| Fang et al, 2012 | ★★★ | ★ | ★★ | good |
| Tu et al, 2017 | ★★ | ★ | ★★★ | fair |
| Zi et al, 2013 | ★★ | ★ | ★★★ | fair |
| Chen et al, 2016 | ★★★ | ★ | ★★ | good |
| Huang et al, 2013 | ★★ | ★ | ★★★ | fair |
| Liu et al, 2020 | ★★ |  | ★★ | poor |
| Xi et al, 2020 | ★★ |  | ★★ | poor |
| Hong et al, 2016 | ★★ | ★ | ★★ | fair |
| Wang et al, 2018 | ★★ | ★ | ★★ | fair |
| Wei et al, 2014 | ★★ | ★ | ★★★ | fair |
| Yan et al, 2016 | ★★ |  | ★★★ | poor |
| Jiang et al, 2018 | ★★ | ★ | ★★★ | fair |
| Hu et al, 2012 | ★★ | ★ | ★★ | fair |
| Nie et al, 2017 | ★★ | ★ | ★★★ | fair |
| Wang et al, 2014 | ★★ | ★ | ★★★ | fair |
| Zhu et al, 2018 | ★★ | ★ | ★★ | fair |
| Jing et al, 2019 | ★★ | ★ | ★★★ | fair |
| Sun et al, 2015 | ★★★ | ★ | ★★★ | good |
| Lin et al, 2014 | ★★ | ★ | ★★ | fair |
| Zhang et al, 2014 | ★★ |  | ★★ | poor |
| Yang et al, 2004 | ★★★ | ★ | ★ | poor |
| Jiang et al, 2011 | ★★ | ★ | ★★ | fair |
| Wang et al, 2014 | ★★ | ★ | ★★ | fair |
| Dong et al, 2013 | ★★ |  | ★★ | poor |
| Zhang et al, 2013 | ★★ | ★ | ★★★ | fair |
| Xie et al, 2016 | ★★ | ★ | ★★ | fair |
| Du et al, 2018 | ★★ |  | ★★ | poor |
| Zhang et al, 2007 | ★★★ | ★ | ★★ | fair |
| Tao et al, 2017 | ★★ | ★ | ★★★ | fair |
| He et al, 2018 | ★★ | ★ | ★★★ | fair |
| Li et al, 2008 | ★★ | ★ | ★★★ | fair |
| Wu et al, 2014 | ★★ | ★ | ★★★ | fair |
| Tu et al, 2017 | ★★ | ★ | ★★★ | fair |
| Tu et al, 2019 | ★★ | ★ | ★★★ | fair |
| Zhang et al, 2003 | ★★★ | ★ | ★★ | good |
| Chen et al, 2020 | ★★ | ★★ | ★★★★ | fair |
