# Supplementary figures and images for: Pooled incidence and case-fatality of acute stroke in Mainland China, Hong Kong, and Macao: A systematic review and meta-analysis
Source: PLoS One. 2022 Jun 27;17(6):e0270554. doi: 10.1371/journal.pone.0270554 (PMC9236238; doi:10.1371/journal.pone.0270554)

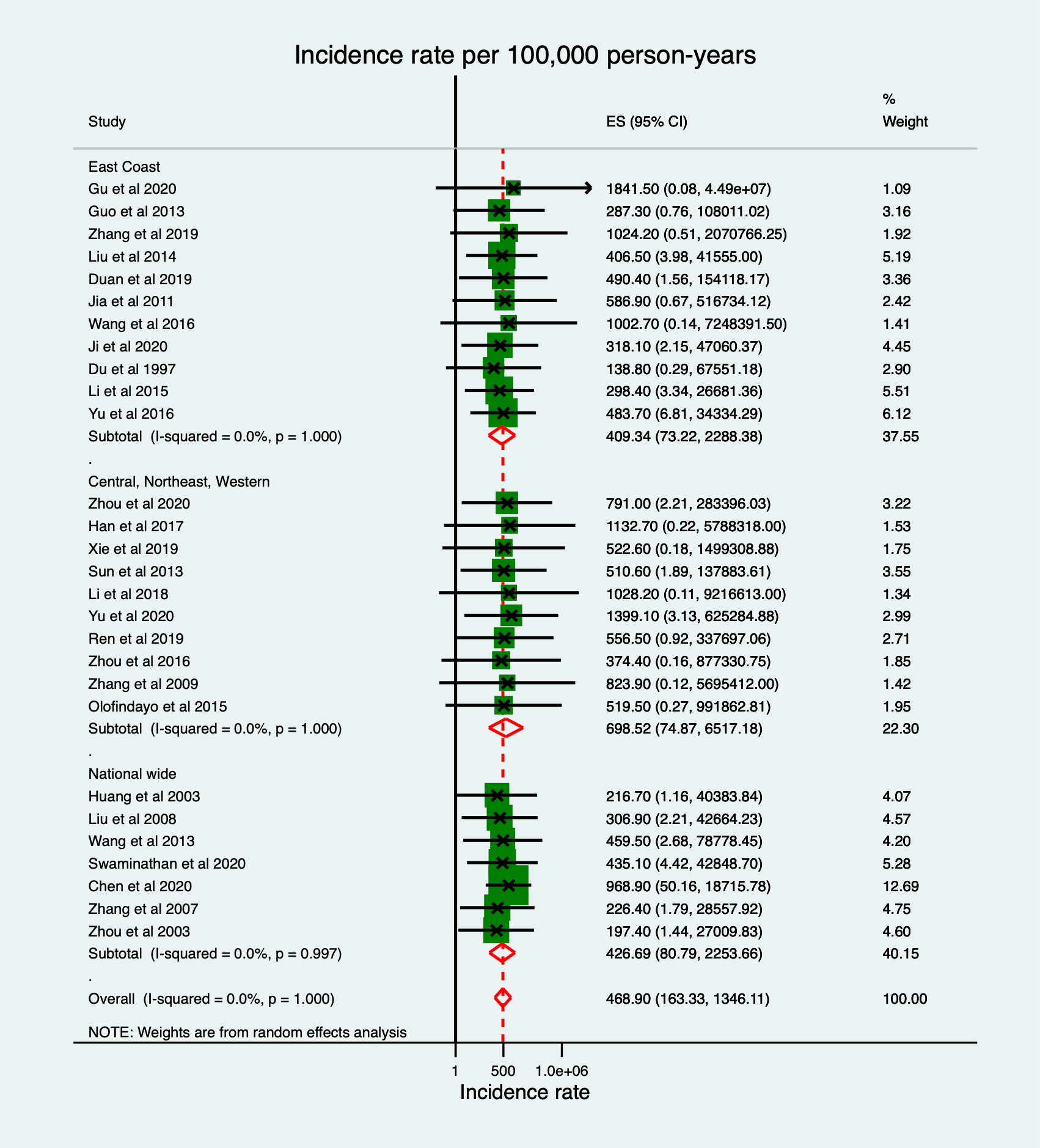

Supplement: S1 Fig — (TIFF) [file pone.0270554.s004.tiff]

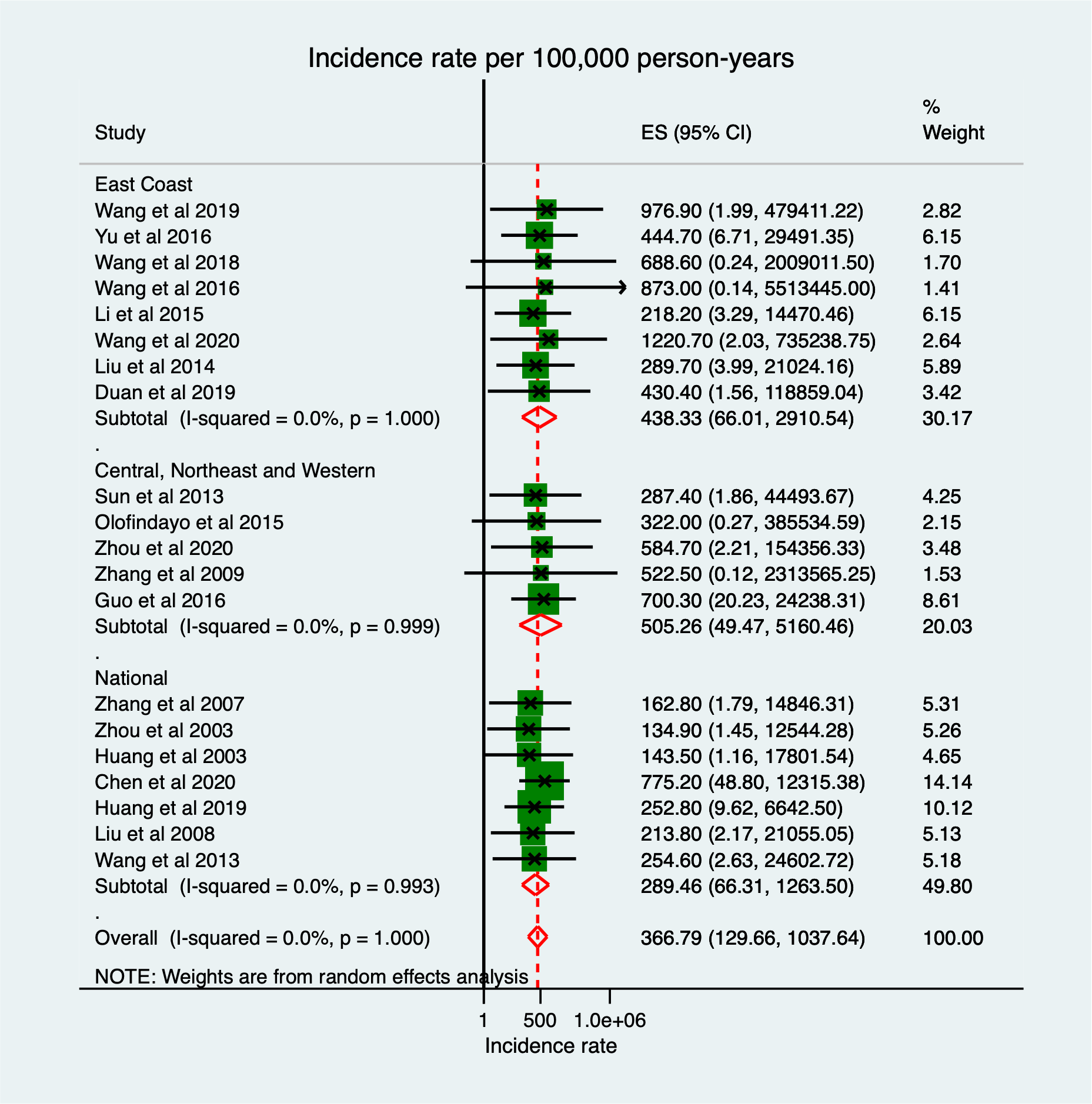

Supplement: S2 Fig — (TIFF) [file pone.0270554.s005.tiff]

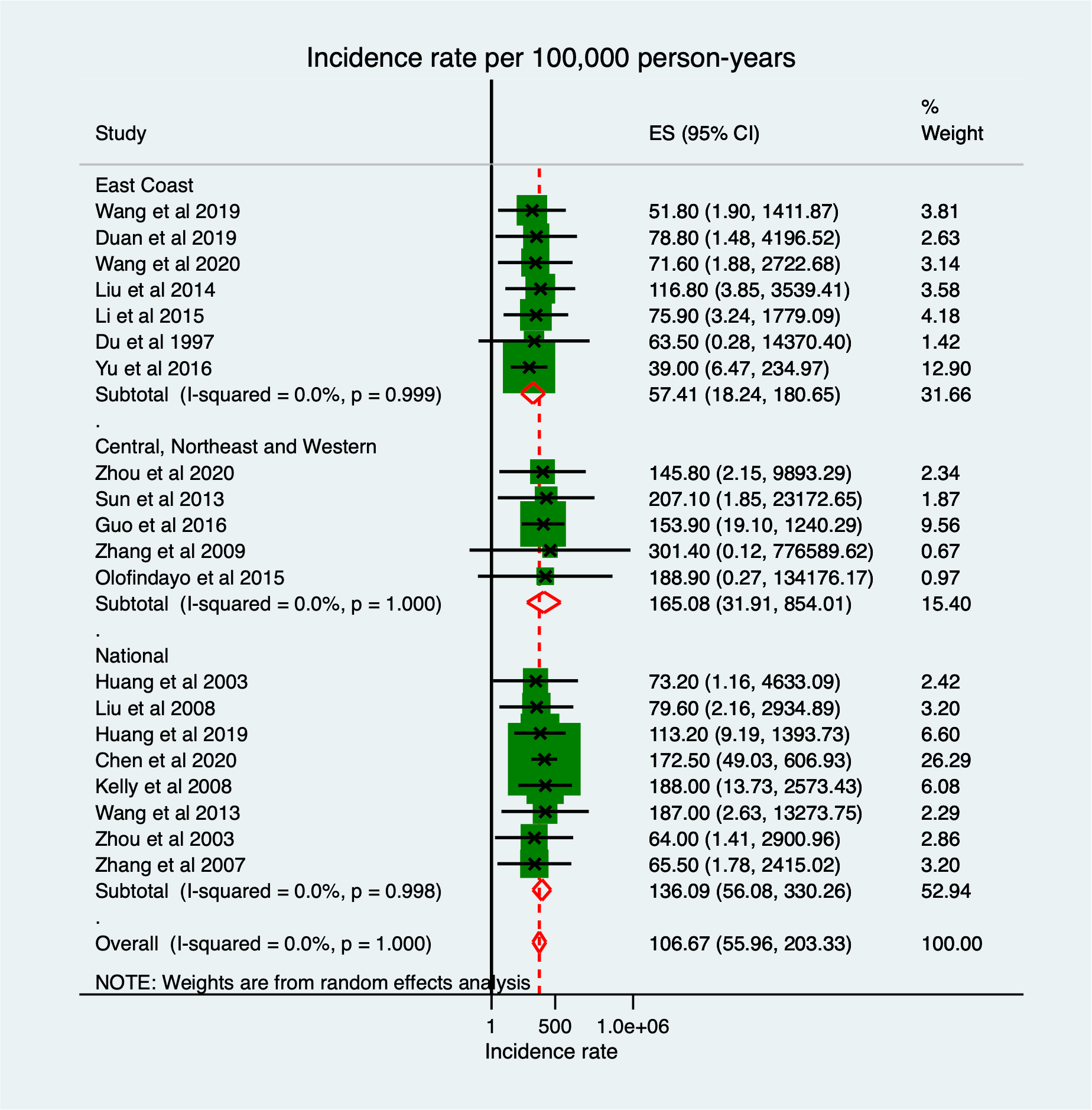

Supplement: S3 Fig — (TIFF) [file pone.0270554.s006.tiff]

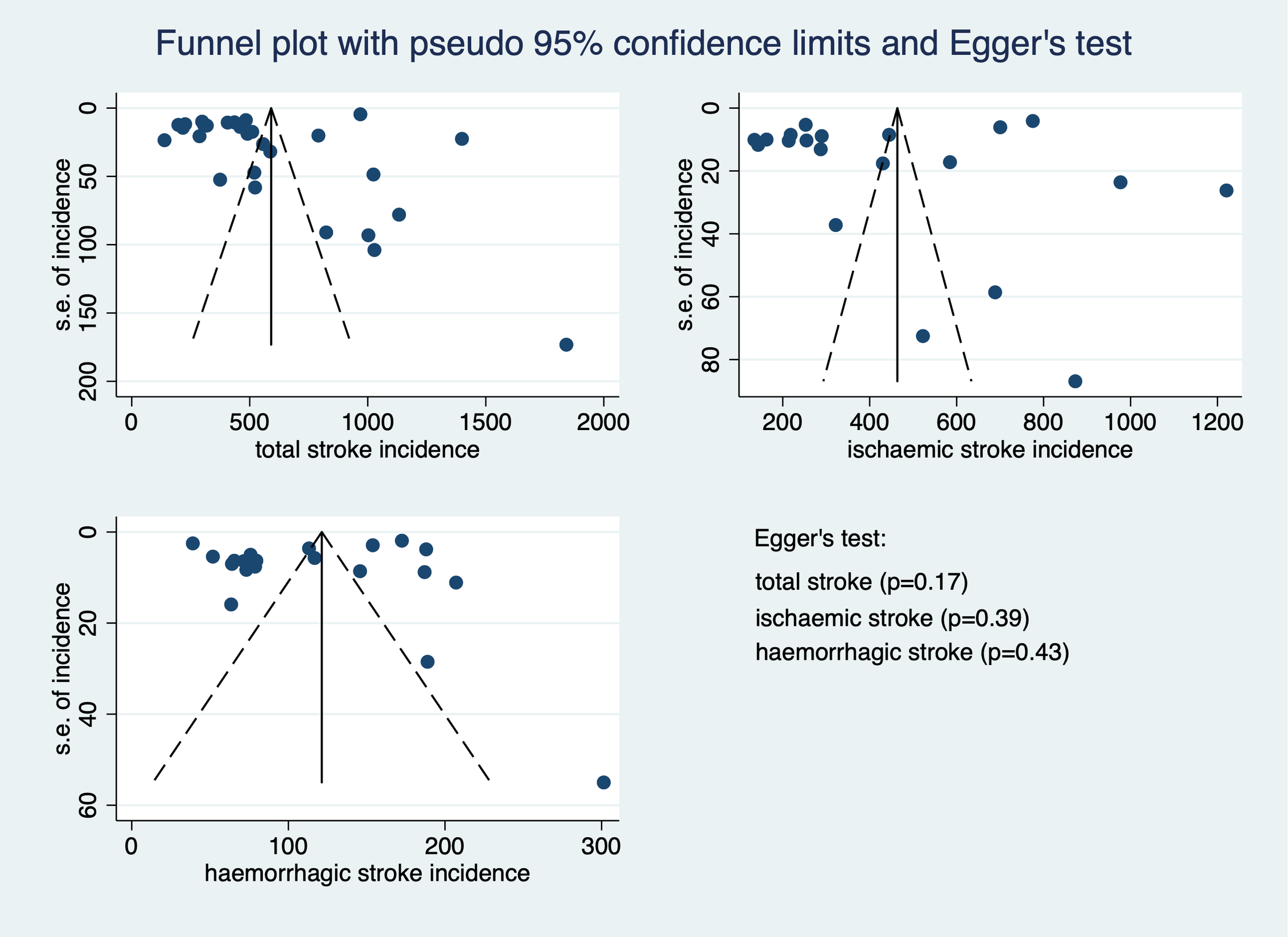

Supplement: S4 Fig — (TIFF) [file pone.0270554.s007.tiff]

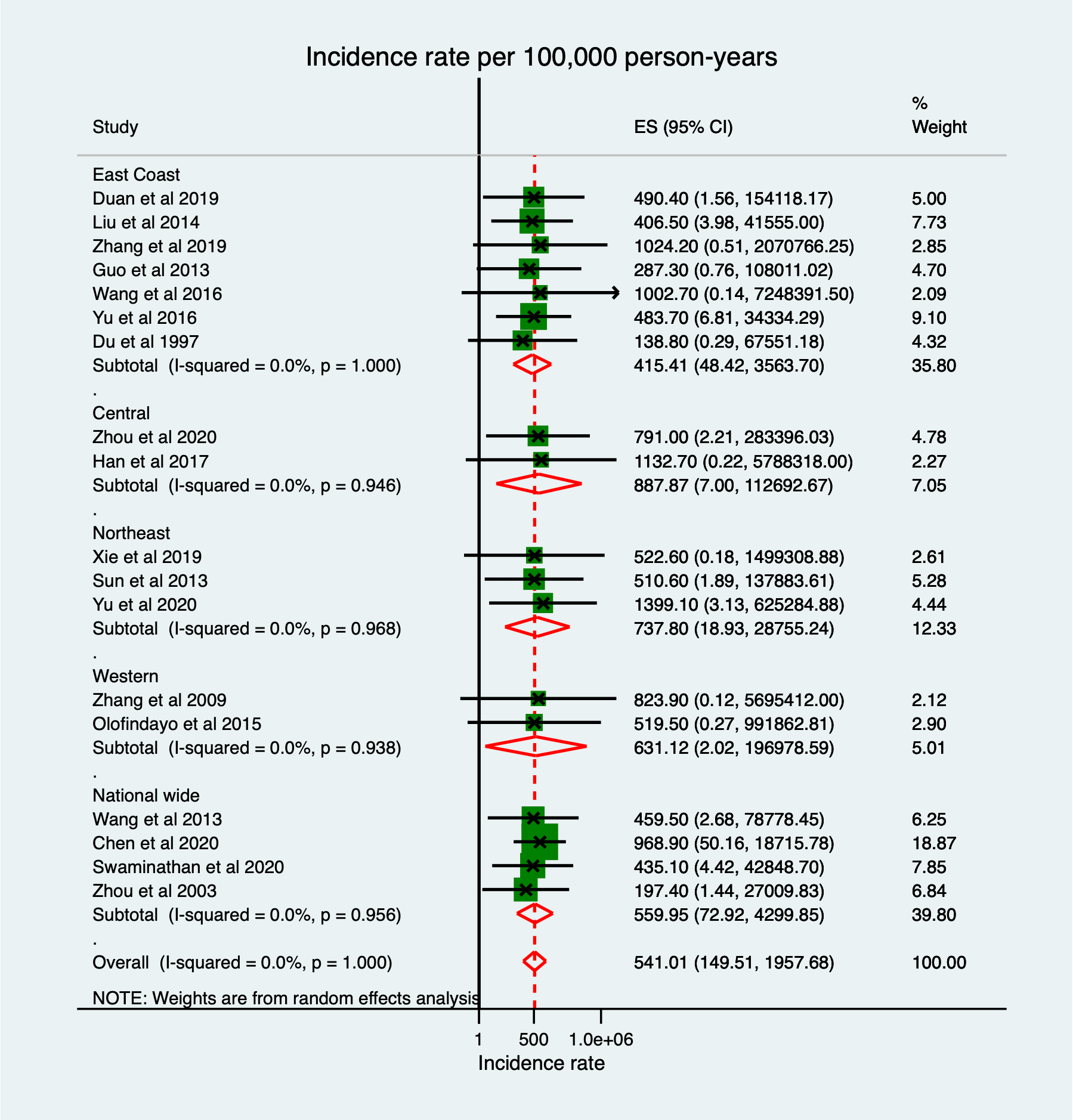

Supplement: S5 Fig — (TIFF) [file pone.0270554.s008.tiff]

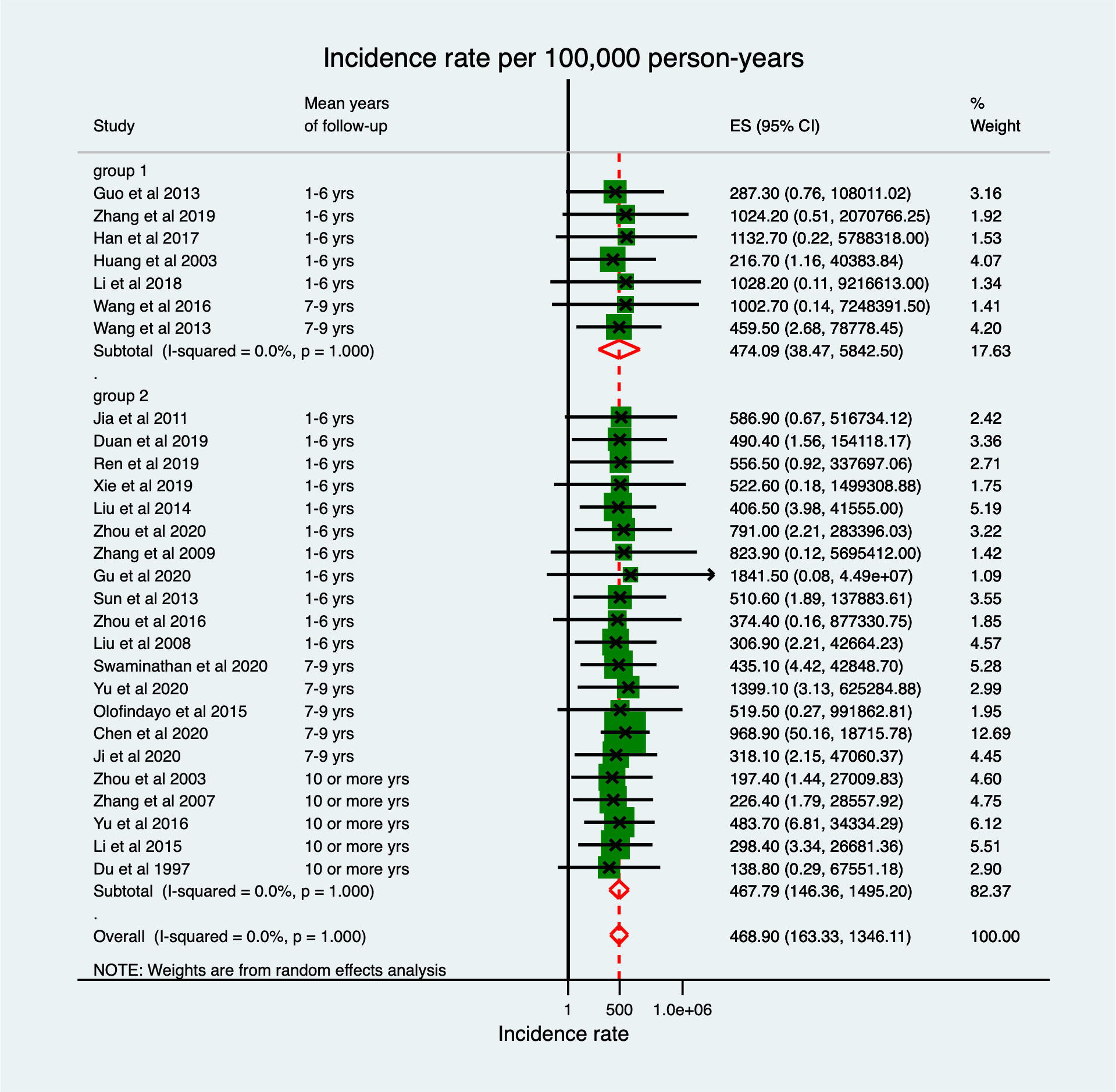

Supplement: S6 Fig — (TIFF) [file pone.0270554.s009.tiff]

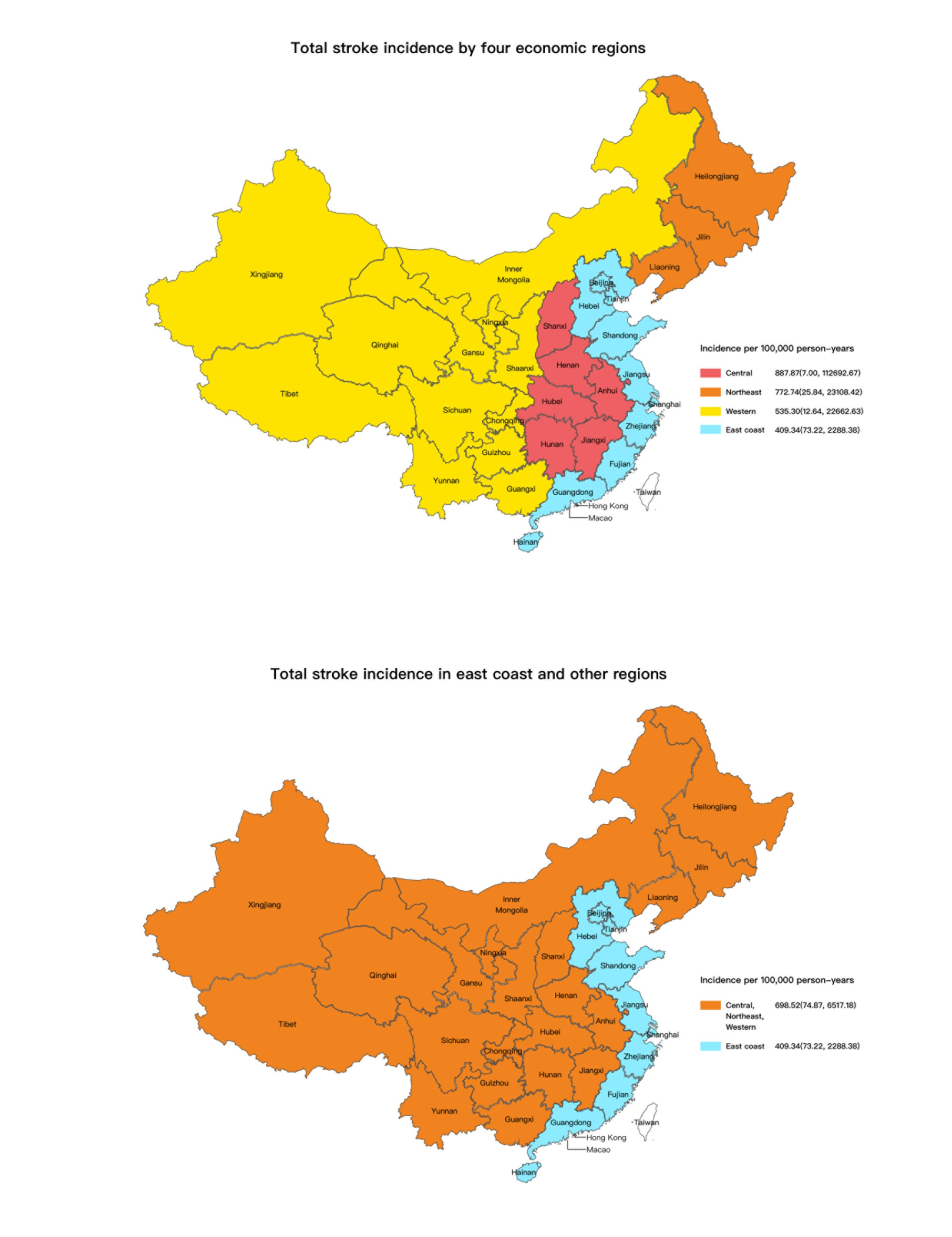

Supplement: S7 Fig — (TIFF) [file pone.0270554.s010.tiff]

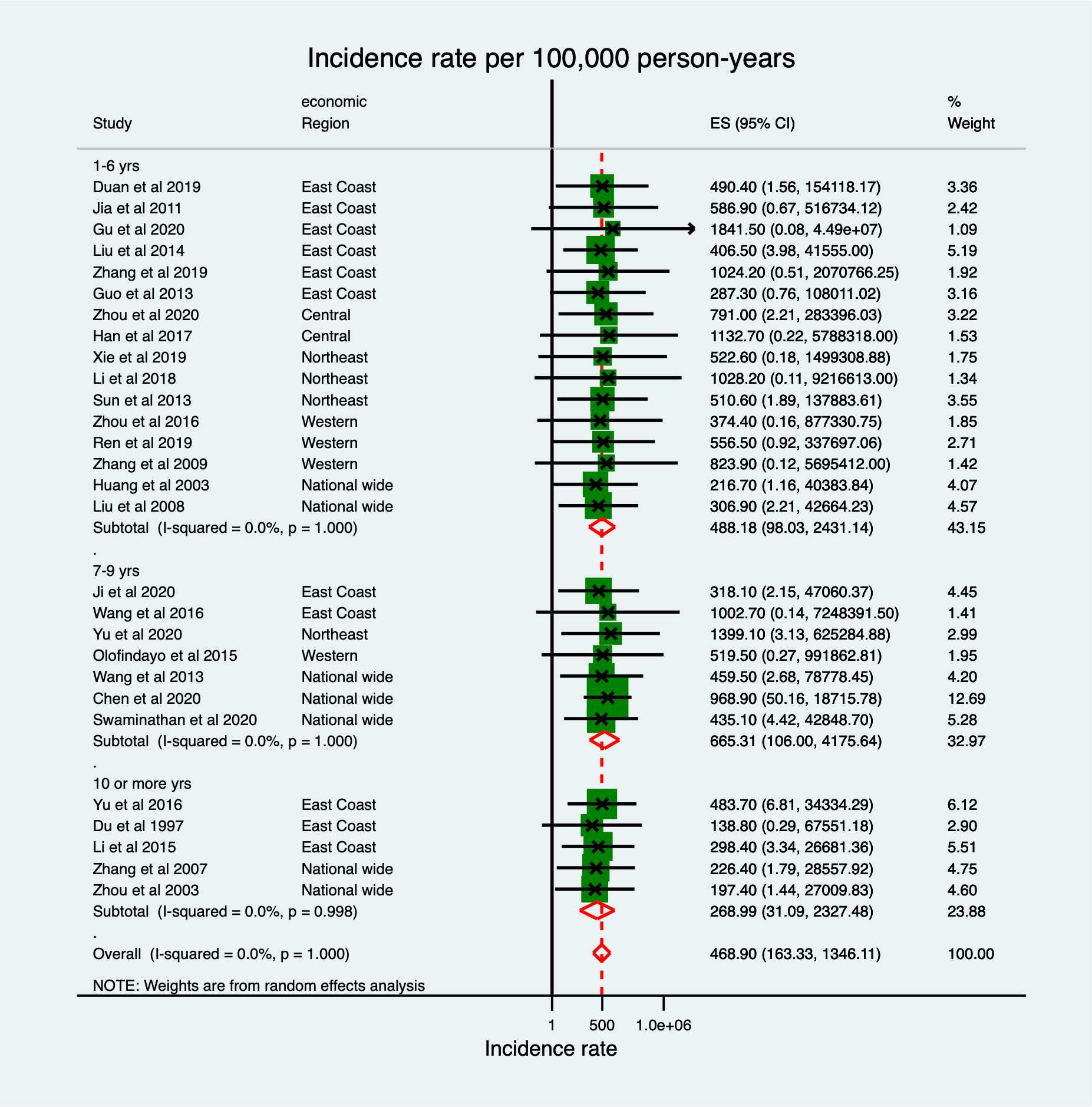

Supplement: S8 Fig — (TIFF) [file pone.0270554.s011.tiff]

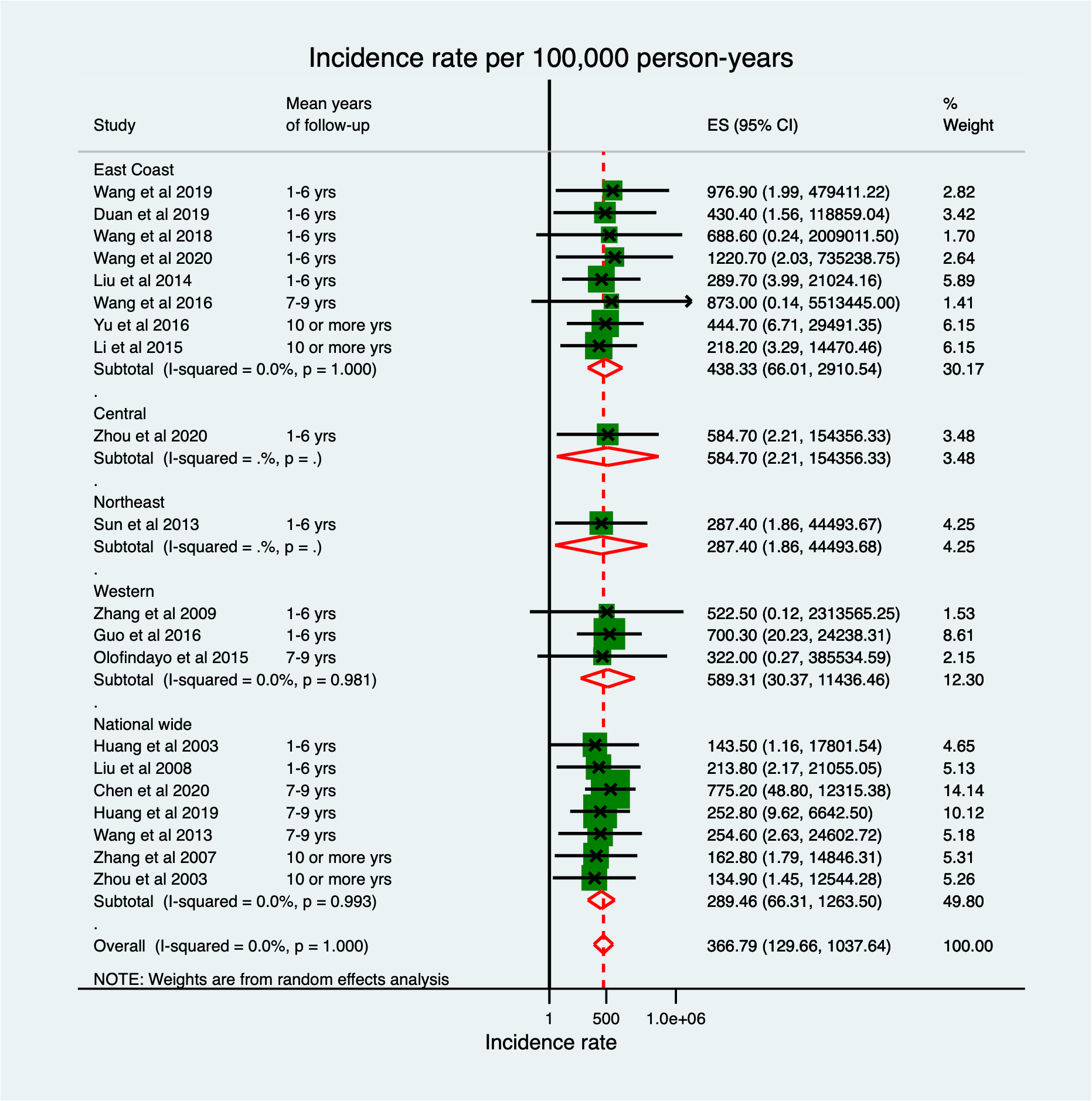

Supplement: S9 Fig — (TIFF) [file pone.0270554.s012.tiff]

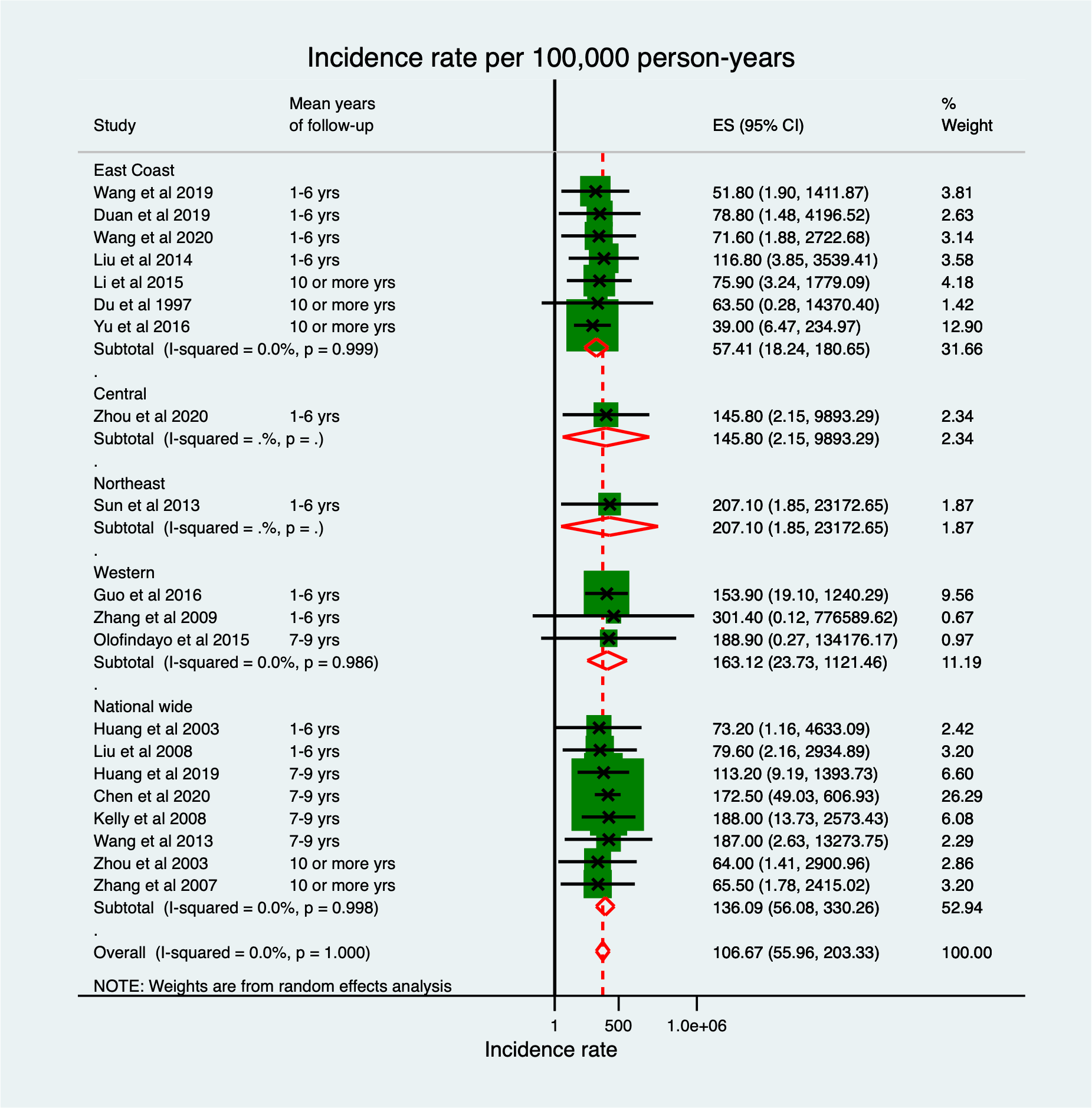

Supplement: S10 Fig — (TIFF) [file pone.0270554.s013.tiff]
