# Supplementary material for: Pooled incidence and case-fatality of acute stroke in Mainland China, Hong Kong, and Macao: A systematic review and meta-analysis
Source: PLoS One. 2022 Jun 27;17(6):e0270554. doi: 10.1371/journal.pone.0270554 (PMC9236238; doi:10.1371/journal.pone.0270554)
Supplement: S1 File — (DOCX) [file pone.0270554.s014.docx]

**S1 File. Included Chinese papers and translations**

杜福昌，王海雁，朱杰，张仕清，钱卫冲，王震震，王敬良. 冠心病与脑卒中发病差异的影响因素. 中华心血管病杂志. 1997;25(1)：16-9.

Du F, Wang H, Zhu J, Zhang S, Qian W, Wang Z, Wang J. Factors influencing the different incidences between coronary heart disease and storke. *Chin J Cardiol*. 1997;25:16-9.

郭吉平，黄久仪，管阳太，陈海英，杨永举， 陶印若，曹奕丰，许英，俞学海. 脑血流动力学改变与脑卒中发病关系的队列研究. 中华预防医学杂志. 2013(4)：323-7.

Guo J, Huang J, Guan Y, Chen H, Yang Y, Tao Y, Cao Y, Xu Y, Yu X. A cohort study on the relationship between cerebrovascular hemodynamic changing and risk of stroke. *Chin J Prev Med*. 2013;323-7.

王小焕，胡锡敏，郭彧，卞铮，李立明，陈铮鸣，李惠梅，李贻磊，符智芳. 海南省成年居民高血压与心脑血管疾病发病关系前瞻性队列研究. 中国公共卫生. 2019;035(8)：986-991.

Wang X, Hu X, Guo Y, Bian Z, Li L, Chen Z, Li H, Li Y, Fu Z. Relationship between hypertension and cardiovascular and cerebrovascular diseases among adult residents in Hainan province: a prospective cohort study. *Chin J Public Health*. 2019;035:986-91.

王小焕，胡锡敏，郭彧，卞铮，李立明，陈铮鸣，李惠梅，李贻磊，符智芳. 海南省成年人吸烟与心血管疾病发病风险的前瞻性研究. 现代预防医学. 2020;47(2): 287-99.

Wang X, Hu X, Guo Y, Bian Z, Li L, Chen Z, Li H, Li Y, Fu Z. Prospective study on tobacco smoking and the risk of cardiovascular disease among adults in Hainan Province. *Modern Prevent Med*. 2020;47:287-99.

任晓宇，史典，张德生，丁皎，李海燕，甘婷，蒲瑞阳，白亚娜，程宁. 金昌队列人群代谢性疾病与脑卒中发病关系前瞻性研究. 中华流行病学杂志. 2019;40(5)：521-5.

Ren X, Shi D, Zhang D, Ding J, Li H, Gan T, Pu R, Bai Y, Cheng N. Prospective study of relationship between metabolic diseases and stroke in Jinchang Cohort. *Chin J Epidemiol*. 2019;40:521-5.

周皖舒，彭年春，时立新，张巧，胡颖，徐淑静，张淼，张松. 踝肱指数的变化与新发不良心脑血管事件的关系. 天津医药. 2016;44(8)：959-62.

Zhou W, Peng N, Shi L, Zhang Q, Hu Y, Xu S, Zhang M, Zhang S. Analysis of the correlation between the change of ABI and new adverse cardiovascular events. *Tianjin Med J*. 2016;44:959-62.

黄久仪，王桂清，沈凤英，曹奕丰，王艳，郭佐，杨永举，樊舜英，徐晓斌，冯春红， 等. 脑血管血液动力学积分与脑卒中风险的队列研究. 中华流行病学杂志. 2003;24(2)：89-93.

Huang J, Wang G, Shen F, Cao Y, Wang Y, Guo Z, Yang Y, Fan S, Xu X, Feng C, et al. A cohort study on cerebral vascular hemodynamics accumulative score and risks of stroke. *Chin J Epidemiol*. 2003;24:89-93.

刘群. 高密度脂蛋白胆固醇与心脑血管病相关性前瞻研究. 中华内科杂志. 2008;47(4):272-6.

Liu Q, Zhao D, Wang W, Liu J, Sun J, Liu J. A prospective study of serum high density lipoprotein cholesterol, cardiovascular and cerebrovascular risk in a Chinese muti-provinces cohort. *Chin J Intern Med*. 2008;47(4):272-6.

张林峰，武阳丰，李莹，李贤，谢高强，赵连成. 中国人群中父母脑卒中史与脑卒中发病关系的前瞻性研究. 中华流行病学杂志. 2007;28(11)：1060-3.

Zhang L, Wu Y, Li Y, Li X, Xie G, Zhao L. A cohort study on parentaI history and risk factors of stroke in Chinese population. *Chin J Epidemiol*. 2007;28(11):1060-3.

周北凡，刘小清，武阳丰，李义和，李莹，麦劲壮，赵连成，饶栩栩，杨军，郭成业，等. 我国中年人群糖尿病和空腹血糖异常对心血管病发病的预测价值. 中华心血管病杂志. 2003;31(3)：226-30.

Zhou B, Liu X, Wu Y, Li Y, Li Y, Mai J, Zhao L, Rao X, Yang J, Guo C, et al. Predictive value of diabetes and impaired fasting glucose to incidence of cardiovascular disease in middle-aged Chinese. *Chin J Cardiol*. 2003;31:226-30.

刘艳艳，张敏，高萍，张志翔，周先举，恽文伟. 伴有中重度脑白质疏松的急性脑梗死患者静脉溶栓疗效分析. 中华医学杂志. 2018;98(13)：998-1002.

Liu Y, Zhang M, Gao P, Zhang Z, Zhou X, Yun W. Influence of intravenous thrombolysis on prognosis of acute ischemic stroke in patients with moderate to severe leukoaraiosis. *Natl Med J China*. 2018;98(13):998-1002.

耿闪，刘娜，孟品，籍牛，孙永安，徐英达，张广慧，何效兵，蔡增林，王蓓，等. 急性缺血性卒中患者血压变异性与认知损害的相关性. 国际脑血管病杂志. 2016;24(11)：992-7.

Geng S, Liu N, Meng P, Ji N, Sun Y, Xu Y, Zhang G, He X, Cai Z, Wang B, et al. Correlation between blood pressure variability and cognitive impairment in patients with acute ischemic stroke. *Int J Cerebrovasc Dis*. 2016;24:992-7.

刘译升，詹艳丽，潘辉，尹家文，胡玥，蔡学礼，刘建仁. 不明原因脑栓塞与心源性脑卒中机械取栓预后的比较. 上海交通大学学报·医学版. 2020;40(9)：1271-6.

Liu Y, Zhan Y, Pan H, Yin J, Hu Y, Cai X, Liu J. Comparison of outcomes after thrombectomy in patients with embolic stroke of undetermined source and cardiogenic stroke. *Journal of shanghai jiao tong university (medical science)*. 2020;40:1271-6.

奚惠娟，吴秋义，陈科春. 重组组织型纤溶酶原激活物静脉溶栓及其时机选择对老年急性脑梗死合并房颤患者预后的影响. 中国老年学杂志. 2020;40(12)：2491-4.

Xi H, Wu Q, Chen Ke. Influence of intravenous rt-PA thrombolysis and its timing selection on prognosis of elderly acute ischaemic stroke patient with comorbid atrial fibrillation. *Chinese Journal of Gerontology*. 2020;40(12):2491-4.

洪雁，安中平. 2479例缺血性脑卒中登记患者三年随访研究. 中华老年心脑血管病杂志. 2016;18(1)：59-63.

Hong Y, An Z. Three-year follow-up of 2479 registered ischemic stroke patients. *Chin J Geriatr Heart Brain Vessel Dis*.2016;18(1):59-63.

蒋敏，顾双双，蔡楠，刘 瑶，张秋灵，王 军，何飞. 血中性粒细胞/淋巴细胞比值对急性脑出血患者临床预后的预测价值. 中国急救医学. 2018;38(12)：1057-61.

Jiang M, Gu S, Cai N, Liu Y, Zhang Q, Wang J, He F. Predictive value of the neutrophil-to-lymphocyte ratio in the clinical prognosis of patients with acute intracerebral hemorrhage. *Chin J Crit Care Dec*. 2018;38:1057-61.

林超，望巧，艾文兵. 脑室内出血临床特征及预后危险因素的前瞻性研究. 中国神经精神疾病杂志. 2014;40(11)：694-7.

Lin C, Wang Q, Ai W. Prospective study on clinical characteristics of intraventricular hemorrhage and risk factors of its prognosis. *Chin J Nerv Ment Dis*. 2014;40(11):694-7.

姜红峰，潘东亚. 急性脑出血的预后影响因素分析. 中华老年心脑血管病杂志. 2011;13(1)：79.

Jiang Hongfeng, Pan Dongya. Influencing factors of prognosis Acute haemorrhagic stroke. *Chin J Geriatr Heart Brain Vessel Dis*. 2011;13(1):79.

杜果，郑波，王庆松，杨翠. 老年大动脉粥样硬化型与心源性栓塞型大血管闭塞脑卒中患者临床预后的对比研究. 中华老年心脑血管病杂志. 2018;20(1)：42-5.

Du G, Zheng B, Wang Q, Yang C. Comparison of clinical outcomes in elderly stroke patients with LAA and those with cardiogenic embolism-induced LVO. *Chin J Geriatr Heart BrainVessel Dis*. 2018;20(1):42-5.
