# Supplementary material for: Pooled incidence and case-fatality of acute stroke in Mainland China, Hong Kong, and Macao: A systematic review and meta-analysis
Source: PLoS One. 2022 Jun 27;17(6):e0270554. doi: 10.1371/journal.pone.0270554 (PMC9236238; doi:10.1371/journal.pone.0270554)
Supplement: Attachment — Submitted filename: Response to reviewers_v6.docx [file pone.0270554.s015.docx]

20^th^ October 2021

Dear Prof. Miguel A. Barboza,

**Re: PONE-D-21-22996 – “Pooled incidence and case-fatality of acute stroke in China: a systematic review and meta-analysis”**

Thank you for the opportunity to revise the manuscript which the reviewer described as *“well structured”* and “*comprehensive”* highlighting the importance of our research project as being “very well discussed”. In line with the editor’s and reviewer’s comments, as requested, we have responded on a point-by-point basis and have changed the manuscript accordingly.

Journal Requirements:

When submitting your revision, we need you to address these additional requirements.

1. Please ensure that your manuscript meets PLOS ONE's style requirements, including those for file naming. The PLOS ONE style templates can be found at 
<https://journals.plos.org/plosone/s/file?id=wjVg/PLOSOne_formatting_sample_main_body.pdf> and 
<https://journals.plos.org/plosone/s/file?id=ba62/PLOSOne_formatting_sample_title_authors_affiliations.pdf>

**Response: The manuscript has been modified against these style requirements.**

2. Please review your reference list to ensure that it is complete and correct. If you have cited papers that have been retracted, please include the rationale for doing so in the manuscript text, or remove these references and replace them with relevant current references. Any changes to the reference list should be mentioned in the rebuttal letter that accompanies your revised manuscript. If you need to cite a retracted article, indicate the article’s retracted status in the References list and also include a citation and full reference for the retraction notice.

**Response: As suggested, the reference list has been checked ensuring it is complete and correct. Retracted papers have not been cited in the manuscript.**

**The reference list has been revised to support the added points in the discussion as suggested by the reviewer. The reference of the accepted protocol of this study has also been added.**

Response to reviewers’ comments

**Reviewer #1:**

Comment 1:

The systematic review and meta-analysis is very well structured, ideas are clear and the writing is concise. I congratulate the author/authors for this. The review of literature is comprehensive and the importance of the research is very well discussed as far as applied perspective and theoretical aspect is concerned. The author/ authors have done good reference to the theoretical fundament for the article.

The tables, figures are helpful to understand the content of the paper.

**Response: We thank the Reviewer for these positive comments.**

Comment 2:

1. The article mentions that there is varying incidence of acute stroke in the the four economic regions with the lowest rates detected in the east coast. This could be because of better healthcare facilities and affordability of population in that region. Increased trends over time in the incidence of TS and IS were observed possibly because of major changes in lifestyle over a period of 4 decades. A three-to-twelve-month fatality following IS increased over time. This could be because of poor rehabilitation, affordability, inadequate follow up and non-compliance with the treatment. This could be because of poor counselling and information to the caregivers about the post stroke care of the patient.

**Response: We thank the Reviewer for these insightful comments. Regional differences in stroke incidence in China have been continuously reported, often explained in the literature as a reflection of different prevalence of major risk factors, including hypertension, smoking, drinking and unhealthy diet. The unbalanced economic development in different regions may have impacted the health care system across different regions. Although populations residing in more developed regions (such as the East coast) may be utilizing health care services more than those residing in less developed regions. Following a comprehensive literature search we could not find any literature to support the notion that inequality in health resources distribution may have contributed to regional differences in stroke incidence. Therefore, we prefer to be more conservative when discussing these differences by only emphasizing the different patterns of risk factors across these regions.**

**As suggested, the possible association between increasing stroke incidence and changing lifestyles is now discussed in the revised manuscript. As mentioned in the original manuscript, improving post-stroke rehabilitation may decrease the three-to-twelve-month fatality following ischaemic stroke (IS). In the revised manuscript we further discussed post stroke rehabilitation and its importance to study outcomes (please see page 19). Affordability is indeed a problem in stroke care in China, which may affect patients’ adherence to secondary stroke prevention medications and access to rehabilitation. The issue of affordability has also been added to this revised manuscript. Non-compliance reflected by nonadherence to medication and insufficient support for the caregivers have similarly been discussed.**

**It is possible that inadequate follow up could have contributed to increasing three-to-twelve-month fatality following IS but we could not support this statement by published literature and therefore we did not add this point to the revised discussion.**

Comment 3
2. This meta-analysis will help in:
a. Reinforcement and concretisation of policies by the policymakers in China to plan according to social and cultural variations of their four economic regions.
b. Planning strategies for primordial, primary and secondary prevention and allocating funds accordingly for infrastructural development.
c. Employing trained workforce in the field of neurosciences for stroke population. Also, developing multi-task force which will comprise of trained healthcare workers, community leaders, private and government sectors and other non-governmental organisations in China.

**Response: We thank the Reviewer for these insightful points, which we added to the revised discussion.**

Yours sincerely,

**Fan He**

La Trobe University, Melbourne
