# Supplementary material for: Pooled incidence and case-fatality of acute stroke in Mainland China, Hong Kong, and Macao: A systematic review and meta-analysis
Source: PLoS One. 2022 Jun 27;17(6):e0270554. doi: 10.1371/journal.pone.0270554 (PMC9236238; doi:10.1371/journal.pone.0270554)
Supplement: Attachment — Submitted filename: Response to reviewer 2_v2_final.docx [file pone.0270554.s016.docx]

24^th^ December 2021

Dear Prof. Miguel A. Barboza,

Re: PONE-D-21-22996R1 – “Pooled incidence and case-fatality of acute stroke in Mainland China, Hong Kong, and Macao: a systematic review and meta-analysis”

Thank you for the opportunity to revise the manuscript. In line with the editor’s and reviewer’s comments, as requested, we have responded on a point-by-point basis and have changed the manuscript accordingly.

Journal Requirements:

1. Please review your reference list to ensure that it is complete and correct. If you have cited papers that have been retracted, please include the rationale for doing so in the manuscript text, or remove these references and replace them with relevant current references. Any changes to the reference list should be mentioned in the rebuttal letter that accompanies your revised manuscript. If you need to cite a retracted article, indicate the article’s retracted status in the References list and also include a citation and full reference for the retraction notice.

***Response: As suggested, the reference list has been checked ensuring it is complete and correct. Retracted papers have not been cited in the manuscript.***

**We slightly revised the title of this manuscript making it consistent with the protocol paper that is currently in print.**

- ***He F, Blackberry I, Yao L, Xie H, Mnatzaganian G. Geographic Disparities in Pooled Stroke Incidence and Case Fatality in Mainland China, Hong Kong and Macao: Protocol for a Systematic Review and Meta-Analysis. JMIR Res Protoc. Forthcoming. doi:10.2196/32566***

Response to reviewers’ comments

Comment 1:

If the authors have adequately addressed your comments raised in a previous round of review and you feel that this manuscript is now acceptable for publication, you may indicate that here to bypass the “Comments to the Author” section, enter your conflict of interest statement in the “Confidential to Editor” section, and submit your "Accept" recommendation.

Reviewer #2: (No Response)

***No Response***

Comment 2:

2. Is the manuscript technically sound, and do the data support the conclusions? The manuscript must describe a technically sound piece of scientific research with data that supports the conclusions. Experiments must have been conducted rigorously, with appropriate controls, replication, and sample sizes. The conclusions must be drawn appropriately based on the data presented.

Reviewer #2: Partly

***Response: We have responded to this comment. Please find our reasoning and responses detailed in the subsequent reviewer’s comments below***

Comment 3
Has the statistical analysis been performed appropriately and rigorously?

Reviewer #2: Yes

***Response: We thank the Reviewer for this positive comment.***

Comment 4

Have the authors made all data underlying the findings in their manuscript fully available?

The PLOS Data policy requires authors to make all data underlying the findings described in their manuscript fully available without restriction, with rare exception (please refer to the Data Availability Statement in the manuscript PDF file). The data should be provided as part of the manuscript or its supporting information, or deposited to a public repository. For example, in addition to summary statistics, the data points behind means, medians and variance measures should be available. If there are restrictions on publicly sharing data—e.g. participant privacy or use of data from a third party—those must be specified.

Reviewer #2: Yes

***Response: We thank the Reviewer for this positive comment.***

Comment 5

Is the manuscript presented in an intelligible fashion and written in standard English?

PLOS ONE does not copyedit accepted manuscripts, so the language in submitted articles must be clear, correct, and unambiguous. Any typographical or grammatical errors should be corrected at revision, so please note any specific errors here.

Reviewer #2: No

***Response: This manuscript has been edited by a native English speaker.***

Comment 6

Please use the space provided to explain your answers to the questions above. You may also include additional comments for the author, including concerns about dual publication, research ethics, or publication ethics. (Please upload your review as an attachment if it exceeds 20,000 characters)

Reviewer #2: I will focus on methods and reporting

***No Response***

Major

1) funnel plots and publication bias tests only make sense in the presence of an intervention, they are meaningless for incidence rates and in this context.

***Response: We respectfully disagree with the reviewer.***

***We used funnel plots together with Egger’s tests to investigate whether the studies included in the systematic review had a biased representation of higher or lower incidences; that is, publication bias.***

***One criticism of meta-analyses is that often the available studies may not be representative of all studies addressing the research question. Publication bias refers to the claim that studies with statistically significant results are more likely to be published than studies without. Publication bias can occur in other instances besides trial study designs (Copper et al 2009). An example of a possible scenario is when a hospital director opposes the publication of results that show very high fatality rates. The funnel plot and Egger’s tests investigated whether our findings on between study heterogeneity was due to publication bias.***

- ***Cooper H et al. The handbook of research synthesis and meta-analysis. 2nd edition. Russel Sage Foundation. New York. 2009***

***Running funnel plots with Egger’s test or other tests that investigate asymmetry is very commonly done in systematic reviews in observational incidence studies. We refer the reviewer to the following publications, which are a few of many that followed this very acceptable statistical method.***

- ***Megan R et al. Systematic review and meta-analysis of the incidence rate of Takayasu arthritis. Rheumatology, 2020; 60(11):4982-4990.***
- ***Makin SDJ, et al. Cognitive impairment after lacunar stroke: systematic review and meta-analysis of incidence, prevalence and comparison with other stroke subtypes. J Neurol Neurosurg Psychiatry. 2013; 84:893-900.***
- ***Hackett ML, et al. Part I: frequency of depression after stroke: an updated systematic review and meta-analysis of observational studies. Int J Stroke. 2014, 9:1017-25.***
- ***Li M et al. Hyperuricemia and risk of stroke: A systematic review and meta-analysis of prospective studies. Atherosclerosis, 2014:265-270.***
- ***Taylor M et al. Longevity of complete dentures: a systematic review and meta-analysis. J Prosthetic Dentistry. 2021; 125:611-617***
- ***Fasugba O et al. Ciprofloxacin resistance in community- and hospital acquired Escherichia coli urinary tract infections: a systematic review and meta-analysis of observational studies. BMC Infect Dis. 2015, 15:545.***
- ***Qin B, et al. Epidemiology of primary Sjögren's syndrome: a systematic review and meta-analysis. Annals of the Rheumatic Diseases 2015;74:1983-1989.***
- ***Meng K, et al. "Incidence of venous thromboembolism during pregnancy and the puerperium: a systematic review and meta-analysis." The Journal of Maternal-Fetal & Neonatal Medicine 28.3 (2015): 245-253.***

2) Language corrections are needed.

***Response: This manuscript has been edited by a native English speaker.***

3) Some justification is needed on the 1990 time limit.

***Response: The decision to exclude studies published before 1991 was made to better capture studies that followed similar diagnostic criteria. Before 1991, clinicians would have diagnosed stroke without brain imaging technologies which could have resulted in the inclusion of false positive cases.*** ***The restriction to 1991 onwards aimed to decrease false positives and to increase the validity of included cases. We have added a clarification in the revised manuscript.***

4) Meta-regression is a stab in the dark usually and is underpowered to detect anything but massive associations (effectively a regression with X observations, where X is the number of available studies). You should discuss this as a major limitation. Even with 60 or 80 studies, it can provide little insight.

***Response: Meta regression offers a systematic approach to explain sources of wide variation in reported findings. In our incidence studies, no heterogeneity was detected and, therefore, there was no need to conduct further analyses. However, in the case-fatality analyses, we conducted meta regression to explain between study variances based on the available study variables.***

***Meta-regression is an extension to subgroup analyses that allows the effect of continuous, as well as categorical characteristics to be investigated, and in principle allows the effects of multiple factors to be investigated simultaneously (although this is rarely possible due to inadequate numbers of studies) (Thompson 2002). According to scientific literature, meta-regression should generally not be considered when there are fewer than 10 to 15 studies in a meta-analysis. Our case-fatality analysis included 39 studies.***

1. ***Cochrane handbook for systematic reviews of interventions.*** [***https://training.cochrane.org/handbook/current***](https://training.cochrane.org/handbook/current)
2. ***Higgins JPT, et al. Measuring inconsistency in meta-analysis. BMJ Clinical Research. 327:557-60.***
3. ***Thompson SG, et al. How should meta-regression analyses be undertaken and interpreted? Statistics in Medicine. 2002; 21:1559-1573***
4. [***https://handbook-5-1.cochrane.org/chapter_9/9_6_4_meta_regression.htm***](https://handbook-5-1.cochrane.org/chapter_9/9_6_4_meta_regression.htm)

***We acknowledge that exploring all sources of heterogeneity is limited as there are many unknown variables that cannot be accounted for. This is always true in all systematic reviews (Copper et al, 2009). Furthermore, the limited number of included studies in the meta-regression add uncertainty in the true sources of heterogeneity. These limitations have been added to the revised manuscript.***

5) Report the confidence intervals for I^2 (calculated using heterogi or metaan in Stata) as argued in http://www.ncbi.nlm.nih.gov/pubmed/17974687. A simple formula exists in the seminal 2002 Higgins paper that proposed I^2.

***Response: The heterogeneity in case-fatality analyses was very high and statistically significant (p<0.001). As requested, using Borenstein and colleagues methods, we have estimated confidence intervals for the statistic I-squared..***

***Borenstein M, et al. Introduction to meta-analysis. John Wiley & Sons, Ltd. UK. 2011***

Minor

1) Add some more info on the methods section in the abstract, was heterogeneity assessed, publication bias assessed (if relevant) etc.

***Response: As suggested, we have revised the abstract to include this.***

2) Clarify that the estimates were back transformed for the forest plots

***Response: The methods we used are stated in the manuscript: “Using the Exact method, the incidence rates of disease were expressed as Poisson means divided by time at risk. The log incidence rates together with their corresponding log standard errors, stratified by the four economic regions, were meta-analysed using DerSimonian and Laird random effects model. Random effects models were also used to estimate the pooled case fatality estimates”.***

3) The axis titles on the forest plots are not clear, reported as percentages when they are supposed to be rates. Currently the methods section and the graphs are not in agreement.

***Response: As shown in Figure 2, the title of axis is incidence rate. While, in Figures 4 and 5, the title of axis is proportion.***

4) How was the random-effect model implemented, i.e. how was heterogeneity estimated? There are numerous ways to do so. Did they use the standard DerSimonian-Laird method? If so, please state so. Also there are better performing methods, for example please see https://www.ncbi.nlm.nih.gov/pubmed/28815652

***Response: This has been stated in the section of “Statistical analysis”: “The log incidence rates together with their corresponding log standard errors, stratified by the four economic regions, were meta-analysed using DerSimonian and Laird random effects model. Random effects models were also used to estimate the pooled case fatality estimates.” We have revised the forest plots showing the case fatalities – now including the weights that were generated by the random effect model.***

5) What Stata commands were used? Again this relates to the point about the methods and the graphs.

***Response: We used metan and metaprop Stata commands for incidence and case fatality studies, respectively. We have added this clarification in Methods.***

6) Clarify the variables included in the meta regression in the methods section.

**Response: The meta-regression models included risk of bias, study setting (community or hospital), follow-up time, smoking status, age, sex, and severity of stroke. This has been clarified in the methods section.**

Yours sincerely,

**Fan He**

La Trobe University, Melbourne
